# Complete Genome Sequence of the Embu Virus Strain SPAn880

**DOI:** 10.1128/genomeA.01155-14

**Published:** 2014-12-04

**Authors:** M. Sofi Ibrahim, Markus Antwerpen, Enrico Georgi, Philipp Vette, Gudrun Zoeller, Hermann Meyer

**Affiliations:** a U.S. Army Research Development and Engineering Command, Edgewood Chemical Biological Center, Aberdeen Proving Ground, Aberdeen, Maryland, USA; b Bundeswehr Institute of Microbiology, Munich, Germany

## Abstract

We report the complete genome sequence of the Embu virus. The genome consists of 185,139 bp and is nearly identical to that of the Cotia virus. This is the first report on the Embu virus genome sequence, which has been considered an unclassified poxvirus until now.

## GENOME ANNOUNCEMENT

Family *Poxviridae* includes two subfamilies, *Chordopoxvirinae* and *Entomopoxvirinae*, which infect vertebrates and insects, respectively. Currently, there are 10 recognized genera of *Chordopoxvirinae*, the most important of which is *Orthopoxvirus* that contains the smallpox variola virus (http://www.ictvonline.org/VirusTaxonomy.asp). The Embu virus is one of a dozen viruses which have not been assigned to a genus in family *Poxviridae* (1, 2). Of these “unassigned” viruses, only the complete genomes of Cotia (3) (accession no. NC_016924), squirrel (accession no. NC_022563), and Yoka (4) (accession no. NC_015960) poxviruses have been published.

The Embu virus was originally isolated from the brain of sentinel suckling white mice in 1962 from the Cotia Field Station of the Instituto Adolfo Lutz, São Paulo, Brazil, and was designated EMBU SPAn880. This virus was considered distinct from the Cotia virus because it failed to react in complement fixation tests. This virus is currently deposited with the American Type Culture Collections as ATCC VR-473. The original isolate was passaged six times in the brain of suckling baby mice in the University of Düsseldorf, Germany. For sequencing, the virus underwent three rounds of propagation in African Green Monkey kidney cells MA104 at 32°C. DNA was extracted using a MagNA pure compact nucleic acid isolation kit. DNA sequencing using Ion Torrent generated 633,692 reads, which were assembled using CLC Genomics Workbench 7.0.4 and DNAStar SeqMan NGen 11 software. Of the total number of reads, 17% were identified as poxvirus-specific when mapped to the Cotia virus, with an average coverage depth of 159. A consensus sequence was generated from these assemblies, and discrepancies were resolved by visual inspection of the raw sequence reads.

The genome of the Embu poxvirus consists of 185,139 nucleotides (nt) and is nearly identical to the Cotia virus strain SPAn232. The central region of the genome contains 157,703 nt, and is flanked by identical, inverted terminal repeats of 13,718 nt at each end. The genome is 76% AT rich. Nine single nucleotide polymorphisms (SNPs) were identified in comparison with the Cotia virus. Five of those SNPs are located in the central region of the genome, two SNPs in the left and two in the right terminal regions. The SNPs in the central region resulted in amino acid changes in two genes (modified ankyrin repeat protein and modified viral late transcription factor). The remaining 183 genes were identical to the Cotia virus.

Not only does the genome sequence of the Embu virus clarify its identity as a Cotia virus, but also it adds to the increasing repertoire of thoroughly characterized poxvirus genomes, which will ultimately contribute to a better understanding of the evolution of *Poxviridae*, one of the most medically important families of viruses.

### Nucleotide sequence accession number.

The complete genome sequence of the Embu virus has been deposited in GenBank under the accession no. KM595078.
